# Tracking Human Immunodeficiency Virus-1 Infection in the Humanized DRAG Mouse Model

**DOI:** 10.3389/fimmu.2017.01405

**Published:** 2017-10-27

**Authors:** Jiae Kim, Kristina K. Peachman, Ousman Jobe, Elaine B. Morrison, Atef Allam, Linda Jagodzinski, Sofia A. Casares, Mangala Rao

**Affiliations:** ^1^United States Military HIV Research Program, Henry M. Jackson Foundation for the Advancement of Military Medicine, Bethesda, MD, United States; ^2^Laboratory of Adjuvant and Antigen Research, United States Military HIV Research Program, Walter Reed Army Institute of Research, Silver Spring, MD, United States; ^3^United States Military HIV Research Program, Department of Laboratory Diagnostics and Monitoring, Walter Reed Army Institute of Research, Silver Spring, MD, United States; ^4^United States Military Malaria Vaccine Program, Naval Medical Research Center, Silver Spring, MD, United States

**Keywords:** human immunodeficiency virus-1, human immunodeficiency virus vaginal transmission, humanized DRAG mouse, RNA, DNA, quantitative RT-PCR

## Abstract

Humanized mice are emerging as an alternative model system to well-established non-human primate (NHP) models for studying human immunodeficiency virus (HIV)-1 biology and pathogenesis. Although both NHP and humanized mice have their own strengths and could never truly reflect the complex human immune system and biology, there are several advantages of using the humanized mice in terms of using primary HIV-1 for infection instead of simian immunodeficiency virus or chimera simian/HIV. Several different types of humanized mice have been developed with varying levels of reconstitution of human CD45^+^ cells. In this study, we utilized humanized Rag1KO.IL2RγcKO.NOD mice expressing HLA class II (DR4) molecule (DRAG mice) infused with HLA-matched hematopoietic stem cells from umbilical cord blood to study early events after HIV-1 infection, since the mucosal tissues of these mice are highly enriched for human lymphocytes and express the receptors and coreceptors needed for HIV-1 entry. We examined the various tissues on days 4, 7, 14, and 21 after an intravaginal administration of a single dose of purified primary HIV-1. Plasma HIV-1 RNA was detected as early as day 7, with 100% of the animals becoming plasma RNA positive by day 21 post-infection. Single cells were isolated from lymph nodes, bone marrow, spleen, gut, female reproductive tissue, and brain and analyzed for gag RNA and strong stop DNA by quantitative (RT)-PCR. Our data demonstrated the presence of HIV-1 viral RNA and DNA in all of the tissues examined and that the virus was replication competent and spread rapidly. Bone marrow, gut, and lymph nodes were viral RNA positive by day 4 post-infection, while other tissues and plasma became positive typically between 7 and 14 days post-infection. Interestingly, the brain was the last tissue to become HIV-1 viral RNA and DNA positive by day 21 post-infection. These data support the notion that humanized DRAG mice could serve as an excellent model for studying the trafficking of HIV-1 to the various tissues, identification of cells harboring the virus, and thus could serve as a model system for HIV-1 pathogenesis and reservoir studies.

## Introduction

Human immunodeficiency virus-1 (HIV-1), the virus that causes acquired immunodeficiency disease is transmitted mainly through the sexual route (1). The early events that occur during HIV-1 sexual transmission and establishment of infection in humans are not completely understood. Insights into HIV-1 transmission in humans have been derived from extensive studies conducted in non-human primate (NHP) models with simian immunodeficiency virus (SIV) (2–4). These NHP studies have highlighted the very early establishment of small populations of founder virus in local areas of entry, early onset of CD4 depletion, and pathological processes in the local areas. These events are followed by an early and a late systemic phase of infection that exert their systemic effects slowly over months to years. Within 7–14 days, the infection became systemic with extensive viral replication and massive CD4 T-cell depletion in the lamina propria (5). An early capture HIV cohort study (RV217) of volunteers in East Africa and Thailand who were at high risk for HIV-1 infection demonstrated that the median peak viremia occurred 13 days after the first plasma sample was positive on nucleic acid testing (6). However, the early HIV events that occur before the plasma becomes HIV-1 RNA positive remain largely unknown.

A major obstacle for studying HIV-1 infection and pathogenesis is the lack of a good animal model. Although extensive studies have been performed in NHP models, these studies have utilized SIV or a chimera simian/HIV (SHIV), which are not the same as HIV-1 (7). Several human–mouse chimeras (humanized mice) have been generated to overcome the limited species tropism of HIV-1. The generation of a mouse with a reconstituted human immune system has enabled the use of humanized mice as a possible model for studying HIV-1 infection. At least 11 different types of humanized mice (8), each with unique characteristics are available. In this study, we utilized a more recently generated strain of humanized mice, the Rag1KO.IL2RγcKO.NOD mice expressing HLA class II-DR4 molecule (DRAG mice) (9–11). These mice were infused with HLA-matched human hematopoietic stem cells from umbilical cord blood and developed a high-reconstitution rate with long-lived functional B and T cells, all four classes of human immunoglobulins, and subclasses of IgG (9). In a previous study, our group has demonstrated that the humanized DRAG mouse model has some important features that correlate better with HIV-1 transmission in humans including high reconstitution of human CD45^+^ cells in the gut and female reproductive tract (FRT) which includes the ovaries, fallopian tubes, uterus, cervix, and the vagina. This reconstitution of human CD45^+^ cells is critical since the gut is an important venue for HIV-1 seeding and systemic spread. A majority of the CD4^+^ T cells present in the DRAG mice also expressed the HIV-1 co-receptor, CCR5. In particular, the CD4^+^ T follicular helper cells in the gut and FRT were highly permissive to HIV-1 infection (10). We also demonstrated that a single intravaginal infection (10,000 TCID_50_; equivalent to 2.54 ng p24) of purified primary HIV-1 resulted in 100% infectivity of humanized DRAG mice (10). The use of primary virus is of increasing importance, especially in light of recent work that indicates that primary viruses behave differently from pseudoviruses and infectious molecular clones (12).

While no animal model can fully mimic the effects of HIV-1 in humans, because of some of the important features mentioned above, the humanized DRAG mouse model is suitable for investigating the early events after HIV-1 infection. Although the presence of SIV/SHIV in the FRT and gut of NHP following an intravaginal challenge are well established and in a separate study it was shown that low levels of viral RNA and DNA were present in distal tissues for several days following low-dose SHIV challenge (13, 14), the trafficking of the virus to the various tissues immediately after infection is still not completely understood. In the present study, we examined various tissues of the humanized DRAG mouse at different time points post-infection following an intravaginal infection with primary HIV-1. We determined how early HIV-1 RNA and DNA could be detected in the various organs post-infection compared with the appearance of the virus in the peripheral blood. Our results show that the earliest detection of viral RNA was in the gut and bone marrow and that the brain was the last organ to become HIV-1 RNA positive. Thus, the humanized DRAG mouse could serve as an excellent model for studying early HIV-1 pathogenesis and presumably also for HIV-1 reservoir studies.

## Materials and Methods

### Mouse Strain

Humanized DRAG mice *[Rag1KO.IL2R*γ*cKO.NOD (“NRG”) strain]* with chimeric transgenes encoding for HLA-DR*0401 [HLA-DRA/HLA-DRB1*0401]) fused to the *I-Ed MHC- II* molecule were generated as previously described (9). Four- to six-week-old DRAG mice were infused with HLA-DR*0401-positive human stem cells (9). Human cell reconstitution was periodically assessed in the peripheral blood samples. The generation of the humanized DRAG mouse is shown schematically in Figure S1 in Supplementary Material. Research was conducted under an approved animal use protocol in an AAALACi accredited facility in compliance with the Animal Welfare Act and other federal statutes and regulations relating to animals and experiments involving animals and adheres to principles stated in the Guide for the Care and Use of Laboratory Animals, NRC Publication, 2011 edition. Human cord blood samples were obtained from the New York Blood Center and were used to reconstitute the mice.

### Intravaginal Infection of Humanized DRAG Mice with HIV-1

Fifty-four female humanized DRAG mice were injected subcutaneously with medroxyprogesterone (2.5 mg per 50 µL per mouse) (Greenstone LLC) 7 days prior to infection. Mice were anesthetized and administered intravaginally with purified primary HIV-1 BaL (10,000 TCID_50_, ~2.54 ng p24) in a total volume of 20 µL as described previously (10). Tissues from 3 animals per time point were collected on days 4, 7, 14, and 21 post-infection for a total of 12 mice. Tissue from two control animals (non-infected) were also collected and processed for RNA and DNA. Plasma viral load over the course of up to 126 days was assessed in the remaining 42 mice. HIV-1 BaL was purified and quantified as described previously (12, 15). HIV-1 BaL was used for infecting the humanized DRAG mice because of its high number of infectious units per milliliter of virus (1.4 × 10^6^ I.U. per mL), as well as a high TCID 50/mL (2.47 × 10^6^), which was necessary to deliver the virus in a small volume into the vaginal vault. In addition, during optimization of the vaginal infection in humanized DRAG mice, we observed a 100% infection rate.

### Isolation of Single Cells from the Gut, FRT, Spleen, Bone Marrow, Brain, and Lymph Nodes

Prior to tissue collection, approximately 1 mL of blood was collected by cardiac puncture. This would be considered as a bleed out since the blood volume for a 25 g mouse is approximately 1.46 mL. A DRAG mouse weighs between 18 and 24 g. Bleed out before tissue collection prevented blood contamination of all the tissues and in particular the brain tissue. The following tissues were obtained from the humanized DRAG mice: gut, FRT, spleen, bone marrow, brain, and inguinal, popliteal, and mesenteric lymph nodes, and placed in 1× HBSS (Ca^++^ and Mg^++^ free), 1× HEPES, 5% FBS (vol/vol) wash buffer on ice. Single cells from the gut were isolated as previously described except collagenase II 1 U/mL (Sigma) was used instead of Collagenase VIII and DNase Type I. Also, the cells were not layered on a Percoll gradient. After centrifugation, the cells were subjected to hCD45^+^ enrichment using anti-CD45 magnetic beads (StemCell Technologies). Cells not bound were removed while the bound cells were subjected to RNA and DNA isolation.

The FRT was processed in a similar manner to the intestinal tissue but was not enriched for hCD45. The fat from the lymph nodes and spleens were removed and single cells were isolated from the lymph nodes and the spleen by pushing them separately through a 70 µm cell strainer using the back of a syringe plunger. Cells were then centrifuged at 1,500 rpm at 4°C for 10 min and stored on ice or frozen until used for RNA and DNA isolation.

For isolation of bone marrow cells, the tips of the femur were cut off and the marrow was flushed into a 70 µm strainer with a syringe and pushed through the strainer using the back of a syringe plunger. After centrifugation, the cells were processed for the isolation of RNA and DNA. The brain tissue was diced into tiny pieces using razor blades and then incubated with collagenase IV (10 mg/100 mL; Life Tech Corp.) in 1× HBSS at 37°C for 90 min on a rotator. The supernatant from the collagenase treatment was placed on a 70 µm strainer and the cells were pushed through the strainer using the back of a syringe plunger. After centrifugation, the brain cells were enriched for hCD45^+^ cells as described above.

Blood (approximately 1 mL) was collected in tubes containing 18 mM EDTA, centrifuged at 3,300 rpm at 4°C and then subjected to RNA and DNA isolation procedures.

### Assessment of Viral Load in the Plasma

Blood samples (30 µL) were collected from humanized DRAG mice pre- and post-infection in tubes containing 18 mM EDTA solution. Following centrifugation at 3,300 rpm for 10 min at 4°C, plasma and the cell pellet were separately stored frozen at −20°C. The viral load in the plasma was determined using the Abbott RealTime HIV-1 Test (Abbott Molecular, Inc.) as previously described (10). The cell pellet was thawed, lysed, and HIV-1 RNA or DNA was extracted and quantified by quantitative real-time (qRT)-PCR. Student’s *t*-test was used to determine if the decrease in viral load on day 42 was significant or not.

### Assessment of HIV-1 Infection in Organs

RNA and DNA were extracted from at least 1 × 10^6^ cells isolated from the harvested organs using the RNeasy Mini Kit and the DNeasy Blood and Tissue kit (Qiagen), respectively. The one-step RT-PCR assay was performed with a Viia7 (Applied Biosystems) using the TaqMan RNA-to-Ct kit (Applied Biosystems). DNA detection qPCR assay was performed using the TaqMan Universal Master Mix II (Applied Biosystems). Two primer/probe sets were used to detect and measure the viral RNA and a housekeeping gene for cellular RNA. The HIV RNA was detected using a primer/probe set for HIV-1 Gag forward: 5′-CATGTTTTCAGCATTATCAGAAGGA-3′, Gag reverse: 5′-TGCTTGATGTCCCCCCACT-3′, Gag probe: 5′-FAM-CCACCCCACAAGATTTAAACACCATGCTAA-BHQ-3′. The primer/probe set used for GAPDH–GAPDH forward: 5′- GAAGGTGAAGGTCGGAGTCAAC-3′, GAPDH reverse: 5′-CAGAGTTAAAAGCAGCCCTGGT-3′, GAPDH probe: 5′-HEX-TTTGGTCGTATTGGGCGCCT-BHQ-3′ (IDT). The reaction mixture (50 µL) contained the following amounts of reagents: 200 ng of total RNA, 1× final concentration of the TaqMan RT-PCR Mix and TaqMan RT Enzyme Mix, 0.2 µM Gag forward primer, 0.2 µM Gag reverse primer, 0.2 µM Gag probe, 0.2 µM GAPDH forward primer, 0.2 µM GAPDH reverse primer, and 0.2 µM GAPDH probe. The amplification reactions were performed using the following program: 48°C for 20 min, 95°C for 10 min (60 cycles of), 95°C for 15 s, and 59°C for 1 min.

Similar to the HIV-1 RNA measurement stated above, HIV-1 DNA was also measured using two primer/probe sets to detect and measure the viral DNA and cellular DNA. The HIV strong stop DNA was detected using the 5′R (5′-AACTAGGGAACCCACTGCTTAA), 3′U5 (5′ TGAGGG ATCTCTAGTTACCAGAGTCA), and R-probe (5′-FAM-CCTCAATAAAGCTTGCCTTGAGTGCTTCAA-BHQ 3′) and the cellular DNA was detected using the same GAPDH primer/probe set mentioned above. The 20 µL reactions contained 200 ng of total DNA, 1× final concentration of the Master Mix, 0.8 µM 5′R (strong stop forward) primer, 0.8 µM 3′U5 (strong stop reverse) primer, 0.25 μM R-probe, 0.8 µM GAPDH forward primer, 0.8 µM GAPDH reverse primer, and 0.25 µM GAPDH probe. The reactions were run using the following program: 95°C for 10 min (60 cycles of), 95°C for 15 s, and 60°C for 1 min. The calculations for determining the RNA or DNA copy number was performed as previously described (12), with the exception of the cell number. The calculated number of cells per reaction was determined and then adjusted using a calculation of 1 ng RNA = 1,000 cells (16). Assay acceptability was contingent on the linear regression *R*^2^ value >0.95 for the viral and cellular RNA and DNA. Cells collected from uninfected control animals did not show any amplification of HIV-1 RNA or DNA positivity and served as negative controls in the study.

### Statistical Analyses

All of the data were graphed and analyzed using GraphPad Prism, version 7.0 (GraphPad Software). Data are represented as mean ± SEM. Student’s *t*-test was utilized to determine statistical significance.

## Results

Sexual transmission of HIV-1 is the most common route of HIV-1 infection. Therefore, humanized DRAG mice were infected vaginally with a single dose (10,000 TCID_50_) of purified primary HIV-1 BaL (subtype B). This dose would be considered as a low to moderate dose based on previous studies where 200,000–700,000 TCID_50_ (20–70-fold higher than our dose) was used (17, 18) for intravaginal infection of humanized mice. However, in two additional studies (19, 20) the intravaginal dose used was 156–3,000 TCID_50_ (3–10-fold lower than our dose).

**Figure 1:**
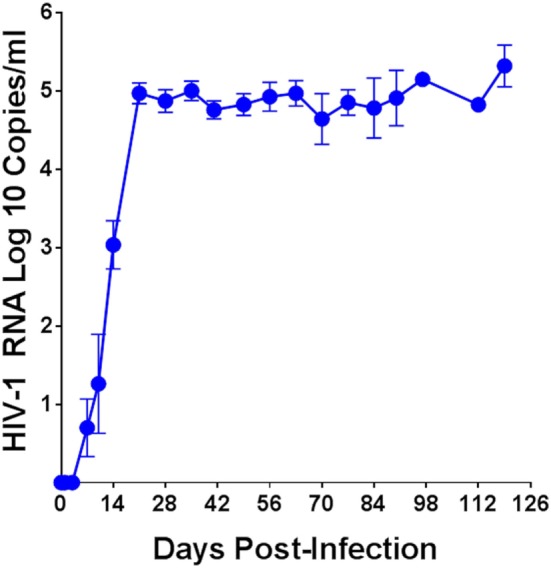
Plasma human immunodeficiency virus (HIV)-1 viral load over time in humanized DRAG mice. Data are the mean ± SEM of 54 humanized DRAG mice infected intravaginally with a single dose of purified primary HIV-1 BaL (10,000 TCID_50_, 2.54 ng p24).

The plasma viral load in humanized DRAG mice was determined over a period of up to 126 days post-HIV-1 infection and the average of 54 mice is shown in Figure 1. Plasma viral loads for each individual humanized DRAG mouse is shown in Figure S2 in Supplementary Material. With a single infection, 89% of the mice became positive by day 14 and 100% of the mice (*n* = 54) became positive by day 21. RNA plasma positivity was detected in some animals as early as day 7 post-infection. Peak viremia was observed on day 21 (3 weeks post-infection), with plasma viral load of 5.0 log 10 copies per milliliter. There was a very slight but insignificant (*p* = 0.29) decrease in the viral load on day 42 with the viral load remaining steady with minimal changes up to day 126 (18 weeks) post-infection.

**Figure 2:**
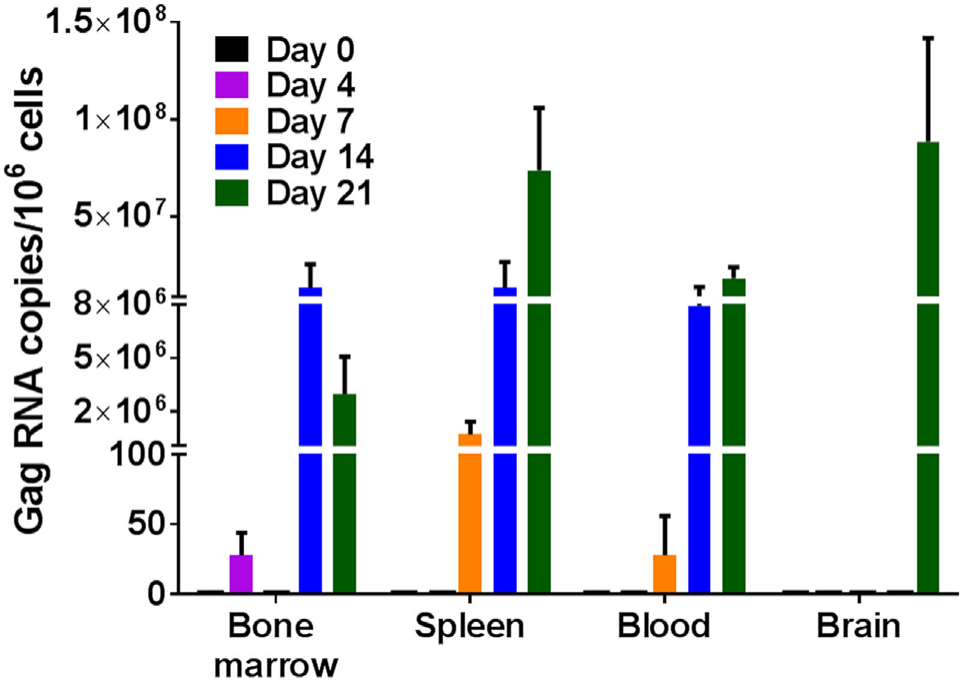
Viral RNA detection in humanized DRAG mice tissues. RNA was isolated from single cell suspensions of bone marrow, spleen, blood, and brain. Viral RNA (gag) and cellular RNA (GAPDH) were detected using quantitative (q) real-time-PCR and quantified using appropriate RNA standards on days 0 (*n* = 2); 4 (*n* = 3); 7 (*n* = 3); 14 (*n* = 3); and 21 (*n* = 3). The data represent the average of triplicate samples ± SEM.

**Figure 3:**
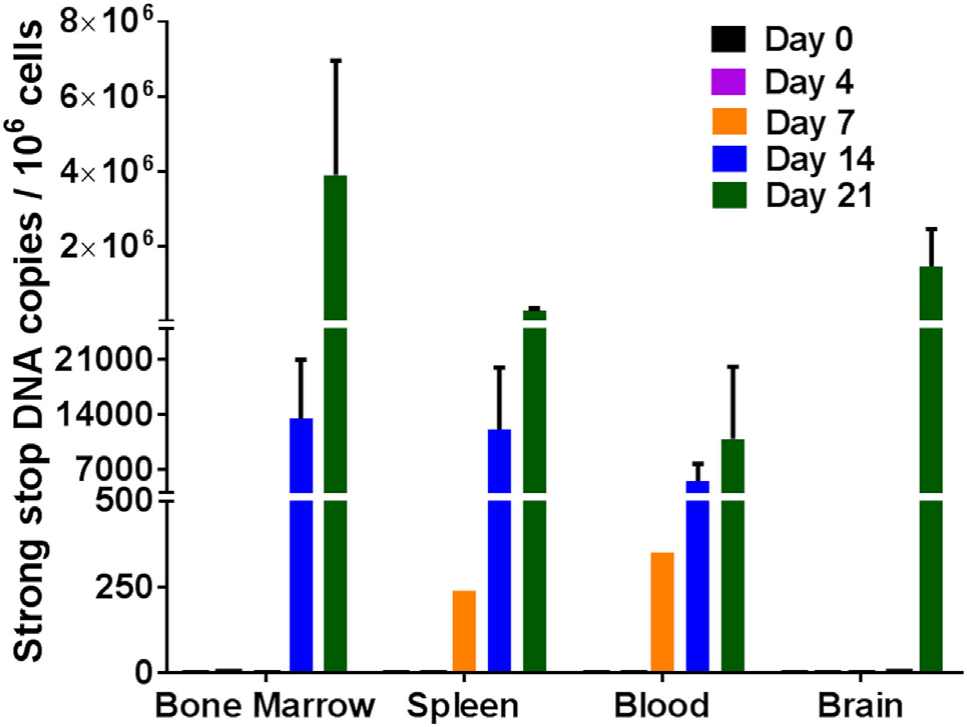
Viral DNA detection in humanized DRAG mice tissues. DNA was isolated from single cell suspensions of bone marrow, spleen, blood, and brain. Viral DNA (strong stop) and cellular DNA (GAPDH) were detected using qPCR and quantified using appropriate DNA standards on days 0 (*n* = 2); 4 (*n* = 3); 7 (*n* = 3); 14 (*n* = 3); and 21 (*n* = 3). The data represent the average of triplicate samples ± SEM.

Single cell suspensions prepared from the bone marrow, spleen, and brain of uninfected humanized DRAG mice or from infected mice on days 4, 7, 14, and 21 post-HIV-1 BaL infection were analyzed for the presence of viral RNA (Figure 2) and DNA (Figure 3). Using qPCR, the number of viral RNA and DNA copies present per million cells was quantified using the appropriate standards and the data are presented in Figures 2 and 3. For blood samples, the EDTA-treated blood was centrifuged, the pelleted cells were lysed, and RNA and DNA were extracted and purified from the cells. As shown in Figure 2, viral RNA was detected as early as day 4 in the bone marrow of two out of three mice, however, no viral RNA was detected on day 7, although low levels of viral RNA were detected in the spleen and blood cells in two out of three mice. By day 14 post-infection, bone marrows of all three mice, spleen cells from two out of three mice, and blood cells from all three mice averaged around eight million gag RNA copies/million cells. The gag RNA copies increased 10-fold in the spleen cells, averaging around 70 million gag RNA copies/million cells by day 21, indicating that the virus was replication competent and spreading to other tissues. In contrast, no viral RNA could be detected in the cells of the brain up to day 14, although the blood, spleen, and bone marrow cells contained actively replicating HIV-1. Unlike the earlier time point, at 21 days post-infection, two out of three mice were positive for the presence of viral RNA in the brain cells. These data demonstrate the prolific nature of the virus and that the brain is probably one of the last organs to become susceptible to HIV-1. It is possible that low levels of HIV-1 may be present at an earlier time point that is undetectable with our assay, which has a lower limit of detection of 10 copies/million. Even though it takes about 21 days to demonstrate the presence of replicating HIV-1 in the brain, in the time frame of infection, it is still relatively rapid.

To further solidify our results that the virus was indeed replicating, strong stop DNA was measured for the same tissue cells as described above for RNA. Similar results were obtained for viral DNA as was observed with viral RNA. Viral DNA was detected in the blood, spleen, and bone marrow cells on days 14 and 21 post-infection (Figure 3). By day 7, approximately 250–400 copies of viral DNA were detected in the spleen and blood tissues. There was an increase in the viral DNA copies by days 14 and 21 post-infection. Similar to the RNA data observed above, no viral DNA was detected in the cells from the brain in any of the mice 14 days post-infection. It was, however, present in two out of three mice at 21 days post-infection. The detection of the viral DNA indicates the presence of replicating virus at these different tissues after intravaginal infection.

Having established that the virus was present and actively replicating in the blood, spleen, bone marrow, and the brain of humanized DRAG mice within 21 days post-infection, we next focused on the FRT, gut, and the lymph nodes, which were of great interest since the gut and the FRT are important organs for seeding and spread of HIV-1 (21–25). Therefore, we examined the cells isolated from the gut, FRT, and lymph nodes at very early time points, on days 4 and 7 post-HIV-1 infection (Table 1). Generally, at this time point, not all of the humanized DRAG mice had detectable viral RNA copies in their plasma and the few that did, had levels that were fairly low.

**Table 1:** Detection of human immunodeficiency virus (HIV)-1 RNA in various tissues.

	FRT	Gut	Lymph nodes
Day 4	0	1	1
Day 7	2	2	3

*Humanized DRAG mice (*n* = 3) were infected intravaginally with a single dose of purified primary HIV-1 BAL (10,000 TCID_50_, 2.54 ng p24). RNA was isolated from female reproductive tract (FRT), gut, and lymph nodes on days 4 and 7 post-infection. The number of mice that were positive for HIV-1 RNA on different days is shown*.

On day 4 post-infection, viral RNA was present in the gut and lymph nodes of one out of three mice and no viral RNA could be detected in the FRT. By day 7 post-infection, viral RNA was detected in the cells of the gut and FRT in two out of three mice. Interestingly, all three mice were positive for viral RNA in cells isolated from the mesenteric and inguinal/popliteal lymph nodes. The presence of viral RNA in the lymph nodes of all three mice suggests that the virus is utilizing the lymphatic system for rapidly spreading to other areas. At day 7, only one mouse had a detectable viral load in the plasma and detectable viral DNA in the blood cell pellet (data not shown) and spleen, while a second mouse showed the presence of viral RNA in the spleen cells, despite an undetectable viral load in the plasma. These data suggest that the virus is probably trafficking to the spleen and bone marrow through the lymphatic system before appearing systemically.

## Discussion

Human immunodeficiency virus-1 replication is limited to only two species: humans and chimpanzees. Rodents cannot be used for HIV-1 vaccine efficacy or transmission studies as their cells lack proper receptor/co-receptor expression along with numerous HIV-1 translational and post-translational replication blocks (26). Thus, the limited species available for *in vivo* HIV-1 studies represents a significant challenge. Furthermore, access to primary human tissue is difficult and requires invasive techniques. NHP and humanized mice have therefore been the models of choice and used extensively to study SIV and HIV infection and pathogenesis. Despite the use of NHP and human tissue biopsies, the early events in SIV or HIV-1 infection are not completely understood (7, 27). In addition to using an appropriate animal model, it is equally important to use the appropriate primary virus. We have recently demonstrated differences in viral capture between primary viruses, pseudoviruses, and infectious molecular clones. Therefore, it is important to use primary HIV-1 propagated in human PBMCs for *in vitro* studies utilizing human tissue biopsies or for *in vivo* studies using humanized mice (12).

There are several models for humanized mice with different strains of mice and different engraftment methods that have been utilized to study HIV-1 infection, including the Hu-PBL-SCID, Hu-SRC-SCID, NSG, NRG, TKO-BLT, and BLT mice (8, 28, 29). In our studies, we have utilized DRAG mice, which has several advantages compared with other strains of humanized mice. Compared with NRG mice, DRAG mice express human HLA-DR4 molecules in cells from spleen, thymus, and bone marrow (9). Previous work has demonstrated that 93% of the humanized DRAG mice were able to reconstitute human T cells mice whereas in humanized NRG mice (RagKO.IL2RgcKO.NOD) which lack the expression of HLA-DR4 molecules, only 36% of the mice were able to reconstitute human T cells (9). This work also indicated that the numbers of human thymic precursors and peripheral human T cells in the T-cell reconstituted DRAG mice were significantly higher when compared with the T-cell reconstituted NRG mice (9). Although the humanized mice models have effective T-cell immune responses, the B-cell functions are not ideal for vaccination and immunization studies. Earlier work by our group has shown that the DRAG mice develop Peyer’s patches (10), while other humanized mice such as NRG, NSG, or BLT mice do not (30). The high level of reconstitution of human T and B cells in the humanized DRAG mice gut, FRT, and spleen, with the majority of CD4^+^ T cells (79–96%) exhibiting a memory phenotype, the ability to generate all four human IgG subclasses, human IgA and IgE, and the ability to elicit specific IgG responses upon immunization makes the humanized DRAG mice an attractive model for pathogenesis and vaccine studies (8–11).

Our present study highlights the use of the humanized DRAG mouse model to determine the presence of HIV-1 in the various organs/tissues at early time points during acute infection following administration of purified primary subtype B HIV-1 (BaL) through the vaginal route. Previous work with humanized mice used infectious molecular clones or primary HIV-1 (19, 31–36). In several of these studies, the mice were injected with these viruses through the intraperitoneal or intravenous routes (31, 32, 34–37). We chose the intravaginal route to infect humanized DRAG mice since the major route of HIV-1 infection in humans is through the sexual route. Our data demonstrate that intravaginal administration of a single dose of purified primary HIV-1 BaL was sufficient for 100% of the mice (*n* = 54) to become HIV-1 RNA plasma positive within 21 days of infection; however, some of the mice became HIV-1 RNA plasma positive as early as 7 days post-infection. The 100% infectivity rate and the high reconstitution of human CD45^+^ cells in the various organs and in particular in the gut and FRT (9, 10) encouraged us to examine the spread of the virus at early time points from the site of infection.

Our study demonstrated the following: (i) HIV-1 viral RNA and DNA with high-copy numbers in some cases, as measured by qRT-PCR, was present in all the tissues examined: bone marrow, spleen, blood, gut, lymph nodes, FRT, and brain; (ii) a progressive increase in the viral copy numbers for both RNA and DNA indicated that the virus was replication competent and spread rapidly; (iii) the earliest detection of HIV-1 RNA was on day 4 in the gut, lymph nodes, and bone marrow; (iv) the brain was the last tissue to become HIV-1 viral RNA positive by day 21.

Even though the brain was the last tissue to become RNA positive, it showed very high viral RNA copies (approximately 100 million) and approximately 2 million viral DNA copies, suggesting that the brain was highly susceptible to HIV-1. The high copy number could be due to a high quantity of HIV-1 trafficking to the brain or due to active HIV-1 replication in this tissue. The presence of HIV-1 in the brain was not due to contamination from the blood as the levels of RNA and DNA copies were 10-fold higher in the brain compared with the blood. Furthermore, while the blood was positive for HIV-1 by day 7, the brain tissue did not become positive for HIV-1 until day 21. Several groups have examined humanized NOD/SCID/IL2Rγ_c_^null^ mouse brain tissue for the presence of HIV-1 after intraperitoneal injections of HIV-1_ADA_ or HIV-infected PHA blasts or after an intravenous infection with HIV-1_MNp_. In the case of the HIV-1_ADA_ virus, DNA was analyzed at day 35 with only one out of the nine mice showing viral RNA in the brain (31). The brain viral RNA was at ~3,000 copies in the mouse with blood viral RNA ranging from 1,000 to 10,000,000 which is in a brain/blood ratio of 3- to 3,000-fold less than what we have observed. Using the intraperitoneal route with HIV-infected blast cells, Singh et al. (38) observed HIV-1-infected cells in the brain as early as 4 days after injection. This study did not determine viral RNA/DNA copies but does support transmission of HIV-1-infected cells into the brain. In the study that utilized HIV-1MN_p_ infection through the intravenous route, viral DNA was analyzed at day 59, significantly later then our 21-day time point and the results showed a threefold higher DNA level in the blood than in the brain (39). The differences between our study and the other studies mentioned above could be due to the route of infection (intraperitoneal or intravenous vs. intravaginal), the virus used, or the time points examined.

The timing of infection in the DRAG mice as well as the trafficking of the virus from the FRT to other organs seem to be in good consensus to what has been reported previously in the NHP/SHIV model (14). After a single intravaginal dose of SHIV-SF162P3 (50,000 TCID_50_), viral RNA was observed only in the vagina and cervix area starting at day 1 or 3 post-infection. At day 7, viral RNA was observed in various organs in the NHP including the mesenteric lymph node, bone marrow, spleen, and brain. By day 10 post-infection, the infection had become systemic with all organs of the three NHPs positive for viral RNA. The variability in DRAG mice on the presence of viral RNA in the various organs at 7 days post-infection was also observed with the NHP model. Although two out of five and four out of five NHPs were positive for viral RNA in the bone marrow and spleen, respectively, the plasma of these animals did not become positive until day 10 post-infection. Similarly, only one out of the five animals in each case was positive for viral DNA in the lymph nodes and spleen.

Our results indicate that HIV-1 infection in the humanized DRAG mouse was a very dynamic and rapid process. As early as day 4 post-infection, viral RNA was detected in the gut, lymph nodes, and bone marrow, although at this time point, the plasma was negative for viral RNA. This would suggest that the virus was utilizing the lymphatic system to spread to the other tissues. It is also important to point out that unlike the spleen and brain cells that contained the highest number of RNA viral copies, the bone marrow contained the highest number of viral DNA copies. This observation may be indicative of bone marrow harboring a viral reservoir at a higher rate than other tissues, although our assay does not distinguish between integrated and non-integrated DNA. This is an interesting observation nonetheless that requires further study. In support of our observation, it has been reported recently that the bone marrow of CD34-NSG humanized mouse was the major tissue site for HIV-1 infection with monocyte-macrophages and dendritic cells being the principal targets following an intraperitoneal infection with a macrophage-tropic virus (HIV-1_ADA_) (31).

Our future work will be directed toward the identification of cells that harbor the virus in the various tissues as well as viral outgrowth assays to determine if the viral RNA negative organs are truly negative for HIV-1. In conclusion, our work demonstrates that the humanized DRAG mouse is an attractive model for studying HIV-1 pathogenesis and establishment of reservoirs because of the high level of reconstitution of human immune cells as well as viral persistence in diverse tissues such as the bone marrow, lymph nodes, and the brain, which could serve as a sanctuary site for HIV-1 to escape the host immune system.

## Ethics Statement

Research was conducted under an approved animal use protocol in an AAALACi accredited facility in compliance with the Animal Welfare Act and other federal statutes and regulations relating to animals and experiments involving animals and adheres to principles stated in the Guide for the Care and Use of Laboratory Animals, NRC Publication, 2011 edition. The protocol was approved by the Institutional Animal Care and Use Committee.

## Author Contributions

MR and JK developed the hypothesis and designed experiments. SC provided humanized DRAG mice. KP and EM infected mice with HIV and collected organs. JK, KP, OJ, and AA isolated single cells from organs. JK performed all RNA and DNA isolation and qPCR. LJ performed plasma RNA viral loads. All authors contributed to the writing and editing of the manuscript.

## Disclaimer

The views expressed are those of the authors and should not be construed to represent the positions of the U.S. Army or the Department of Defense.

## Conflict of Interest Statement

The authors declare that the research was conducted in the absence of any commercial or financial relationships that could be construed as a potential conflict of interest.

## Supplementary Material

Figure S1 Schematic representation of the generation of the human-immune-system DRAG mice. Procedure for generating the humanized DRAG mice as described in Danner et al. (9).

Figure S2 Plasma HIV-1 viral loads over time in individual humanized DRAG mice. A total of 54 humanized DRAG mice were infected intravaginally with a single dose of purified primary HIV-1 BaL (10,000 TCID_50_, 2.54 ng p24).
