# Supplementary figures and images for: Tracking Human Immunodeficiency Virus-1 Infection in the Humanized DRAG Mouse Model
Source: Front Immunol. 2017 Oct 27;8:1405. doi: 10.3389/fimmu.2017.01405 (PMC5663722; doi:10.3389/fimmu.2017.01405)

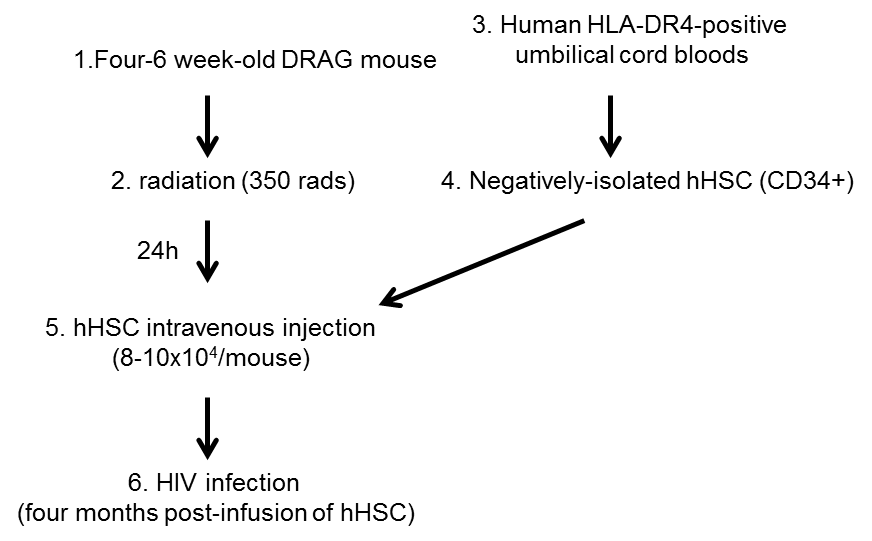

Supplement: Figure S1 — Schematic representation of the generation of the human-immune-system DRAG mice. Procedure for generating the humanized DRAG mice as described in Danner et al. (9). [file Image_1.TIF]

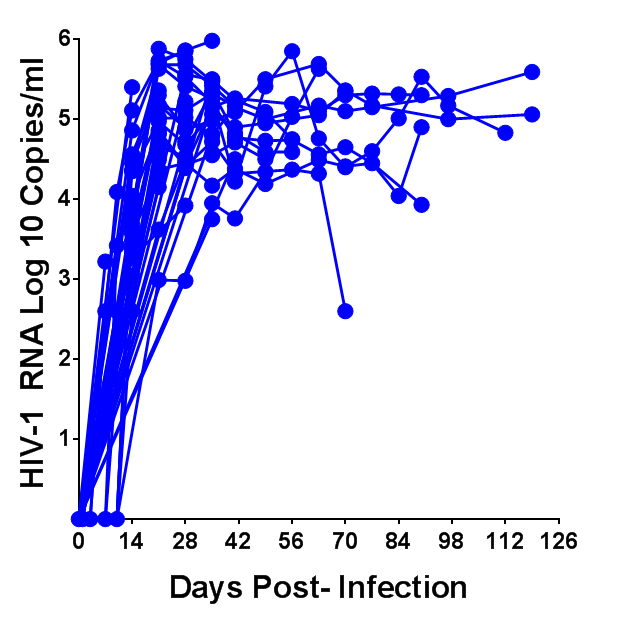

Supplement: Figure S2 — Plasma HIV-1 viral loads over time in individual humanized DRAG mice. A total of 54 humanized DRAG mice were infected intravaginally with a single dose of purified primary HIV-1 BaL (10,000 TCID50, 2.54 ng p24). [file Image_2.TIF]
